# High-resolution Annotated Dataset of *Girvanella* Boundstone Microfacies from the Xiannüdong Formation, China

**DOI:** 10.1038/s41597-026-06958-1

**Published:** 2026-03-05

**Authors:** SoonYoung Choi, DaeCheol Kim, Jongsun Hong, ByungGil Lee, JongDae Do, ChangHwan Kim, ChangWook Lee

**Affiliations:** 1 Dokdo Research Center, East Sea Research Institute, Korea Institute of Ocean Science and Technology, Uljin, 36315 Republic of Korea; 2 Division of Science Education, Kangwon National University, Chuncheon, 24341 Republic of Korea; 3 Department of Geology, Kangwon National University, Chuncheon, 24341 Republic of Korea; 4 East Sea Environment Research Center, East Sea Research Institute, Korea Institute of Ocean Science and Technology, Uljin, 36315 Republic of Korea

**Keywords:** Sedimentology, Stratigraphy

## Abstract

The calcimicrobial crust–cement boundstone in the Xiannüdong Formation of the Cambrian Series 2 is a distinctive reef type that reflects the evolution of late Precambrian stromatolite structures. This study provides a high-resolution dataset by segmenting large slab images of *Girvanella*-based reefs into 114 × 114 pixel tiles and annotating microfacies components through a point-counting-based automated labeling.The dataset includes PNG images and corresponding CSV files and can be used as training data for deep learning-based classification of carbonate microfacies. It contributes to research on the evolution of ancient marine ecosystems and the structure of early carbonate platforms.

## Background & Summary

Cambrian Series 2 is marked by a rapid increase in the complexity of marine ecosystems and the emergence of various biogenic–inorganic composite reef structures^1–3^. The Xiannüdong Formation in the Yangtze Block of China particularly preserves well-developed calcimicrobial boundstone structures composed of *Girvanella* and marine cement, which are considered to inherit the structure of late Precambrian stromatolites^4,5^. *Girvanella* is interpreted as the calcified sheath of filamentous cyanobacteria and is frequently found as domal crusts in Cambrian carbonate rocks. These microstructures are considered important geological evidence for understanding the interaction between biologically-induced calcification and marine chemical environments^1^. Recently, there has been increasing demand for high-quality datasets suitable for deep learning-based classification of carbonate microfacies in various geological contexts such as the Jafnayn Formation in Oman, Cambrian microbial reefs, and Ediacaran carbonate systems^6–10^. This study offers a robust training dataset by manually annotating each microcomponent (*Girvanella* crust, peloid, bioclast, etc.) from real slab images of the Xiannüdong Formation.

The slab images and thin sections used to construct this dataset were derived from calcimicrobial crust–cement boundstones of the Xiannüdong Formation (Cambrian Series 2), collected from the Shatan section (Sichuan Province) in the Yangtze Block, South China. These samples represent reefal deposits formed in shallow-subtidal, inner-platform to near-shoal environments under moderate- to high-energy conditions. Detailed descriptions of the stratigraphic framework, sample locality, and paleoenvironmental interpretations are provided in the original geological study from which these slab materials were obtained^6^.

## Methods

To develop a high-resolution dataset of carbonate microfacies information from the Xiannüdong Formation suitable for deep learning analysis, we designed a Python-based automated tiling and annotation workflow. The full workflow includes: (1) sampling and imaging of slab specimens, (2) tile segmentation based on grid overlays, (3) class determination using annotation masks, (4) dataset organization, and (5) validation and visualization.

**Fig. 1:**
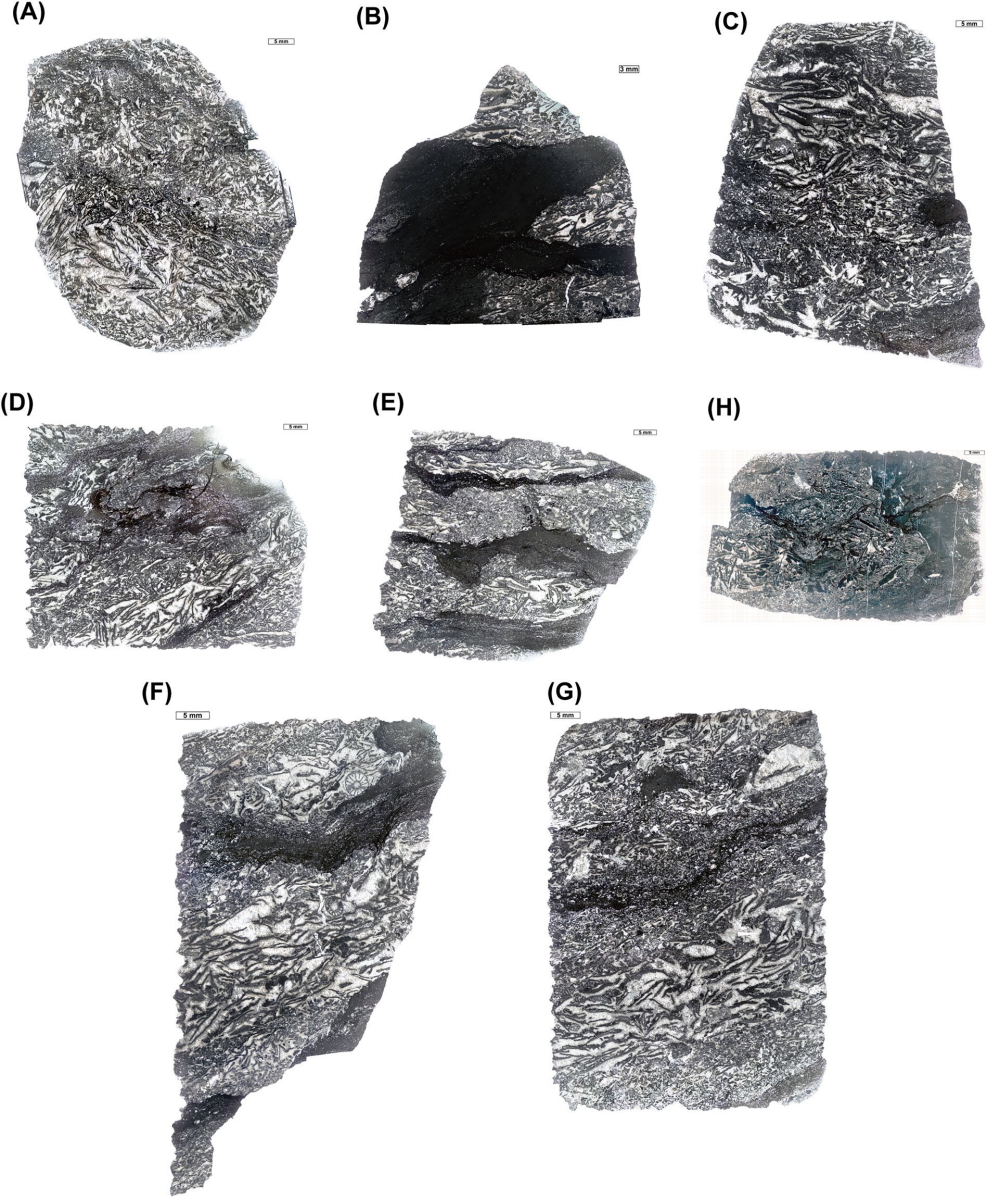
Representative thin section images from the Xiannüdong Formation. Each sample was captured using a digital microscope with a 5 mm scale bar. The images illustrate the morphological diversity and microbial structures of the calcimicrobial boundstone. (**A**) 20150212 HXND 2-3, (**B**) Unit 1 1-1(1,1), (**C**) Unit 1 1-2(1,1), (**D**) Unit 1 1-2(2,1), (**E**) Unit 1 1-2(3,1), (**F**) Unit 1 1-2(4,1), (**G**) Unit 1 1-2(4,2), (**H**) Unit 1 1-5(3,1).

**Fig. 2:**
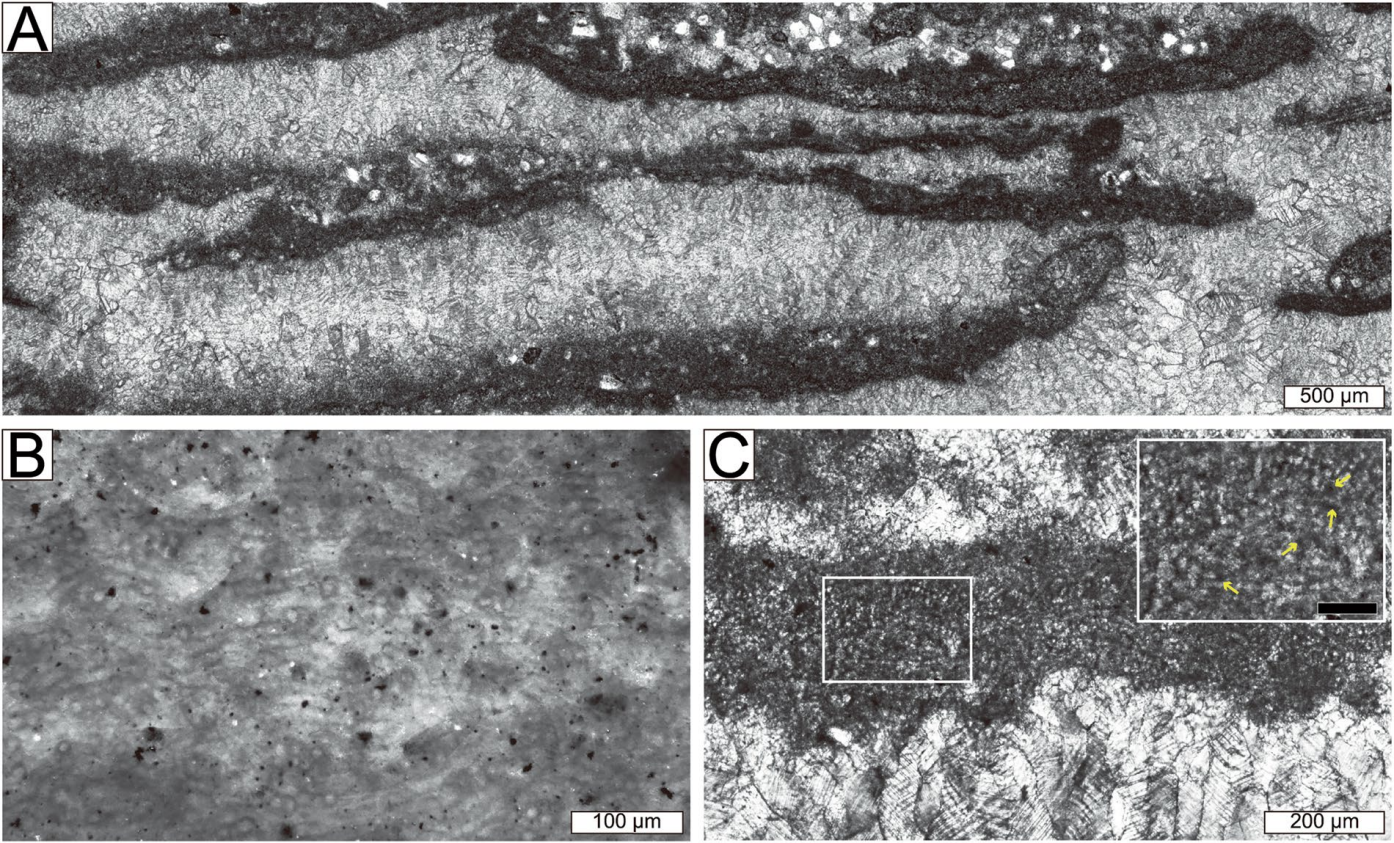
Photomicrographs of reef-building components in *Girvanella* crust–cement boundstone. (**A**) Arcuate to tabular micritic *Girvanella* crusts, oriented horizontally to vertically. (**B**) Well-preserved crust showing irregularly entangled *Girvanella*, visualized using the white-card technique. (**C**) Poorly preserved crust in which tubules appear as densely packed threads.

**Fig. 3:**
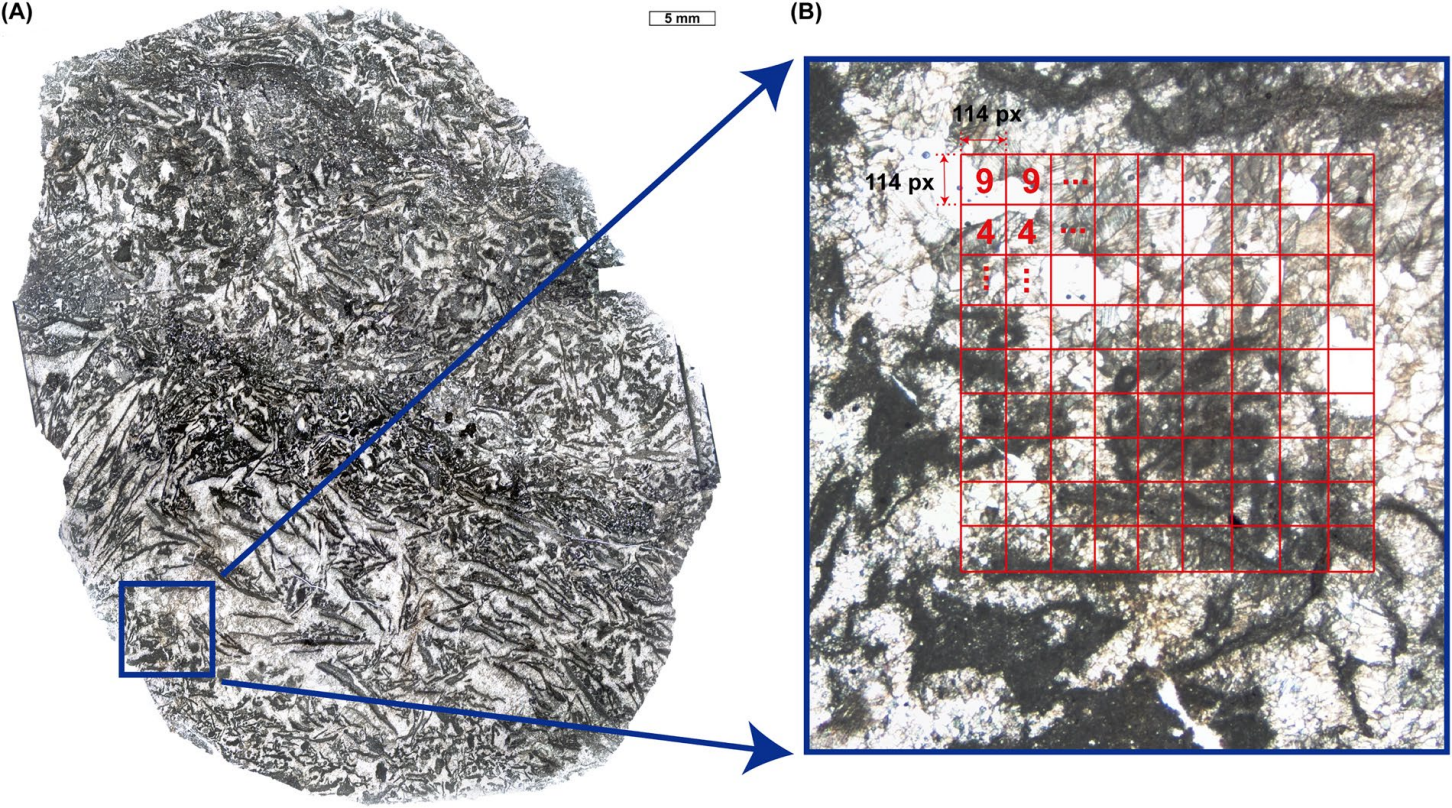
Tile resolution and labeling procedure (example). (**A**) A representative thin section image is divided into tiles of 114 × 114 pixels using a superimposed grid. (**B**) Close-up view illustrating class assignment based on the sum of pixel values in the green channel of number.png, where each value corresponds to a predefined microfacies class. *Note: The red-colored class numbers are for visual reference only; actual label IDs are encoded as integer values in the green channel. The tile resolution (114 × 114 px) is consistent with the main Methods section*.

**Fig. 4:**
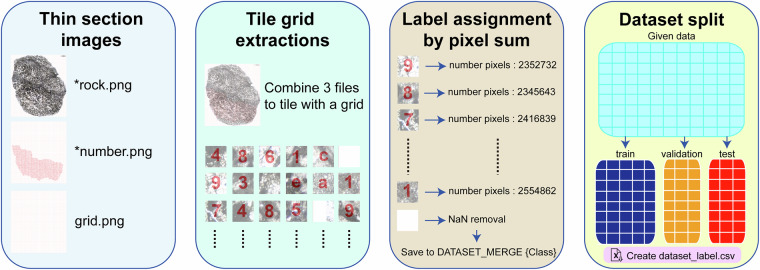
Workflow for tile-based dataset construction. The pipeline includes (1) input of annotated thin section images (rock.png, number.png, grid.png), (2) tile generation using a defined grid, (3) label assignment via green channel pixel summation, and (4) stratified splitting into training, validation, and test sets with corresponding dataset_labels.csv generation.

Samples were collected from 28 carbonate slabs in the Xiannüdong Formation at the Shatan section (Sichuan Province), South China. Representative thin-section slab images are shown in Fig. 1. Whole-slab images were obtained using a polarizing microscope and high-resolution digital camera with a resolution of approximately 0.01 mm/pixel. Seven slabs were selected for image processing, segmented into 114 × 114 pixel tiles using grid overlays created in Adobe Illustrator. In addition to routine petrographic observation, selected thin sections were examined using a white-card technique to enhance contrast and facilitate recognition of filamentous microstructures in variably preserved microbial crusts. Representative examples illustrating preservation variability and diagnostic filamentous features consistent with *Girvanella* are shown in Fig. 2. Class annotations were applied using grid.png and number.png files. The slab images were organized into three key formats: grid.png, rock.png, and number.png (Figs. 3 and 4). Each slab image was segmented into 114 × 114 pixel tiles based on the predefined grid (Fig. 3). Class labels were determined using a point-counting technique, where the total pixel sum of the green channel in number.png was used to identify microfacies classes. Each predefined pixel sum corresponded to a specific class^11^. The number.png files served as annotation masks distinguishing microstructural components, created through manual labeling (Fig. 3). The Python script detects horizontal and vertical grid lines in grid.png to divide rock.png into 114 × 114 pixel tiles. In the number.png image, class numbers are visually overlaid in red for human readability; however, this visual annotation serves only as a reference. The actual class labeling is performed based on the integer values stored in the green (G) channel at the pixel level. This dual-layer design separates visual interpretation from algorithmic processing, where the red channel is intended for GUI-based inspection and the green channel functions as metadata for computational labeling. Each tile is assigned a class label by calculating the total sum of green-channel pixel values within the corresponding region of the number.png label image. This sum is matched against a predefined set of reference values, each corresponding to a specific class ID. If no match is found, the tile is excluded from the dataset. This rule-based assignment enables consistent and fully automated labeling across all thin section samples. This approach fully automates the labeling process and ensures consistent label assignment based on tile position. For example, intraclast(a) is assigned class 0, and bioclast(6 + c) is grouped under class 6, resulting in a total of 10 classes. Tiles containing undefined or unlabeled regions (NaN) are excluded from the tiling process. Cement subtypes were not differentiated in the current annotation scheme; incorporating cement-type subdivisions would require additional petrographic criteria and is considered a potential direction for future dataset expansion.

Tiles containing undefined or missing values were excluded from the final dataset. All valid tiles were saved in the directory structure DATASET_MERGE/{class_number}. To assess class distribution, the number of tiles per class was visualized. The complete dataset was then automatically split into training (80%), validation (10%), and test (10%) subsets using stratified sampling to maintain class balance (Fig. 4). The tiles were stored under dataset_train/, dataset_val/, and dataset_test/ directories. The dataset serves as a standardized classification system for deep learning and machine learning-based image analysis^7^. In addition, a metadata file (dataset_labels.csv) listing all image paths and corresponding class IDs is provided to ensure reproducibility. This automated processing pipeline improves reproducibility and enables the construction of large-scale training datasets annotated under consistent rules across multiple slab versions. The workflow is summarized in Fig. 4, and detailed instructions are provided in the **Supplementary Information**.

## Data Records

The dataset is available at Figshare^12^ and is organized into two main directories:

- /images/: Contains all tile images in 114 × 114 pixel PNG format. Files are named using numeric identifiers and are extracted from thin section slides with grid-based tiling. These images serve as the input data for training, validation, and testing of machine learning models. Within /images, tiles are organized into dataset_train/, dataset_val/, and dataset_test/ subsets, and each subset contains class-specific folders (0–9).
- /labels/: Contains a single CSV file (dataset_labels.csv) that provides class annotations for each image tile. The file includes three columns: filepath (relative path to the tile image), label (microfacies class ID), and subset (assigned to train, validation, or test set). This format ensures consistent annotation and reproducibility across the entire dataset.

On Figshare, the dataset is provided as structured directories rather than a single bulk archive, allowing users to directly access individual image tiles and associated annotation files. This directory-based organization improves file-level discoverability and facilitates reuse in machine learning workflows in accordance with FAIR principles. The dataset structure is designed to support seamless integration with machine learning pipelines while maintaining clear traceability between images, annotations, and data partitions.

## Technical Validation

**Fig. 5:**
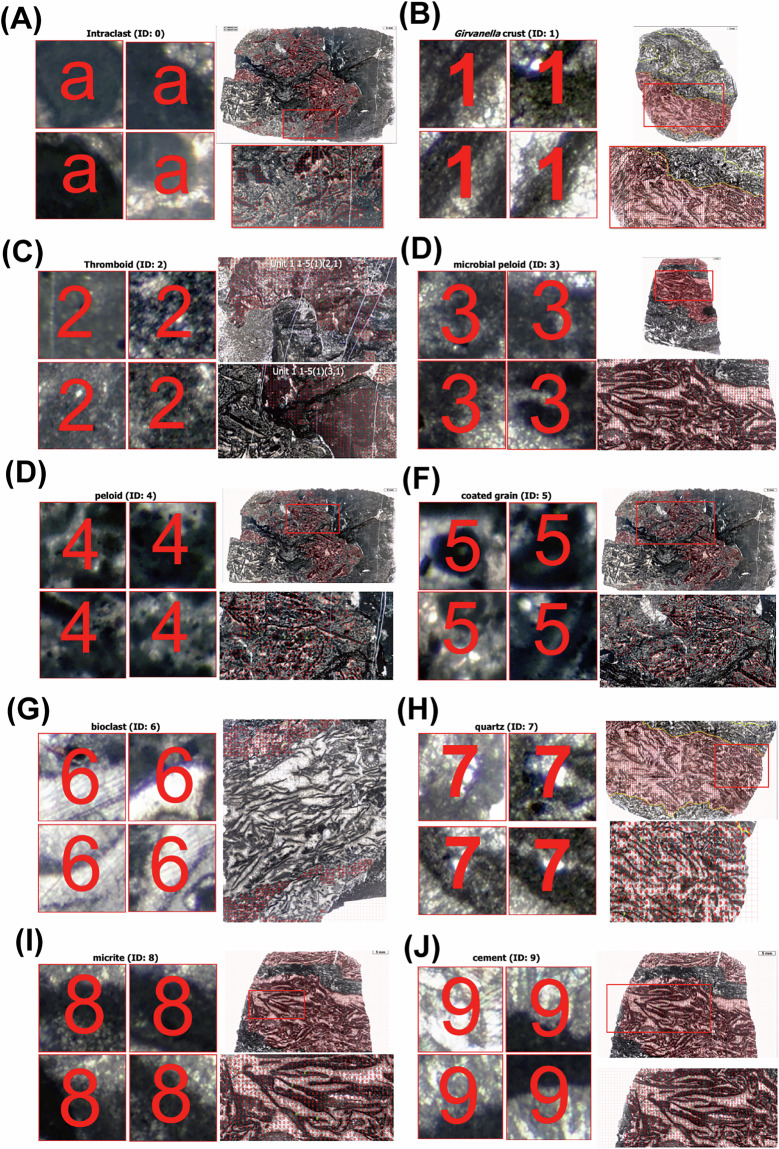
Representative photomicrographs and extracted tile examples for each annotated class in the *Girvanella* crust–cement boundstone dataset. For each class (ID 0–9), four tile images used for deep learning training are presented alongside whole-slab source images with red annotations indicating tile extraction areas. Class names and their original slab sources are as follows: (**A**) intraclast (Unit 1 1-5(1)(3,1)); (**B**) *Girvanella* crust (20150212 HXND 2-3); (**C**) thromboid (Unit 1 1-5(1)(2,1) & (3,1)); (**D**) microbial peloid (Unit 1 1-2(1,1)); (**E**) peloid (Unit 1 1-5(1)(3,1)); (**F**) coated grain (Unit 1 1-5(1)(3,1)); (**G**) bioclast (Unit 1 1-2(4,1)); (**H**) quartz (20150212 HXND 2-3); (**I**) micrite (Unit 1 1-2(1,1)); (**J**) cement (Unit 1 1-2(1,1)). Full label information and extraction sources are also summarized in Table 2.

**Fig. 6:**
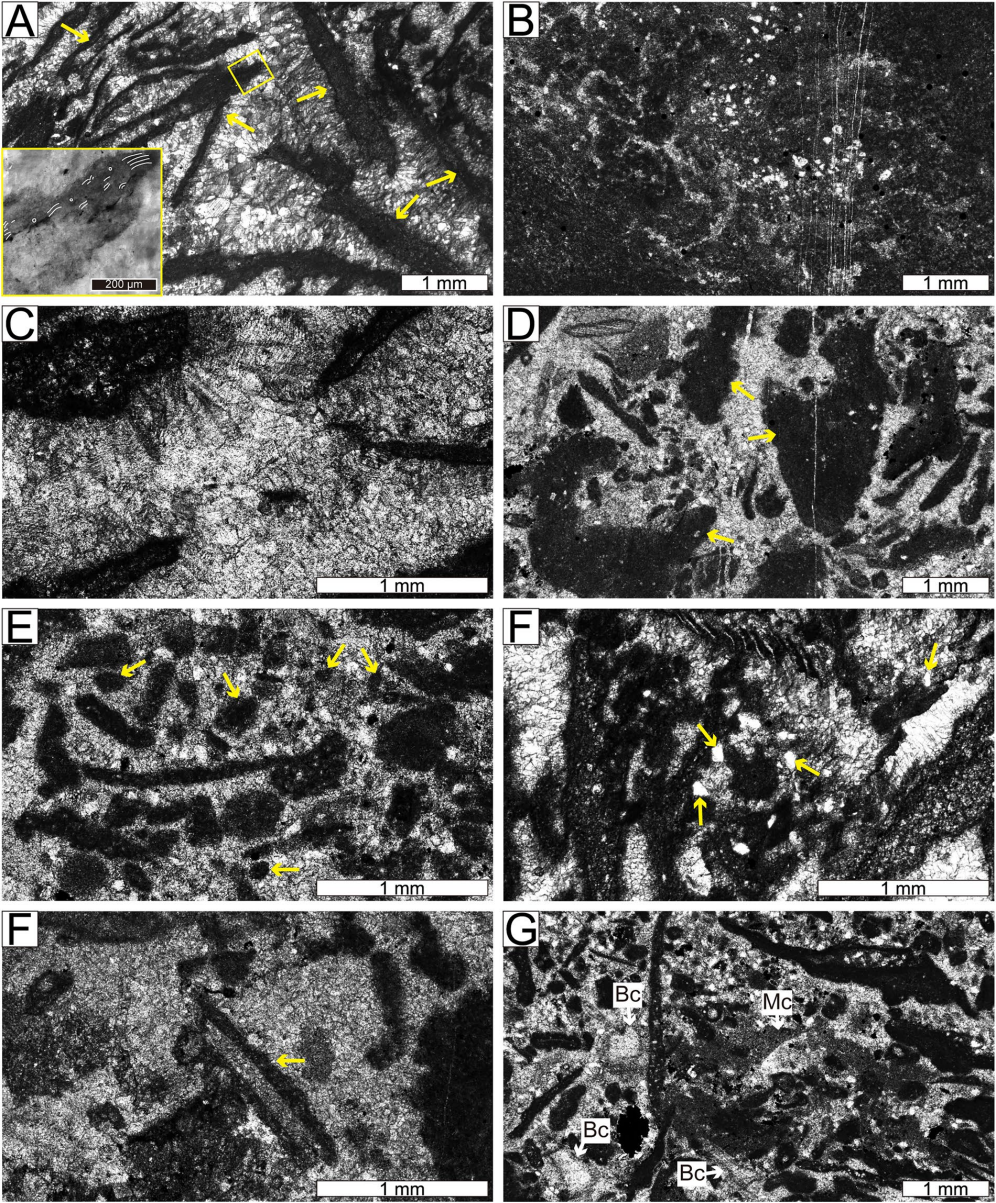
Photomicrographs of components in *Girvanella* crust–cement boundstone and surrounding peloid–intraclast packstone to grainstone. (**A**) arcuate to tabular micritic *Girvanella* crusts (arrows), oriented horizontally to vertically. The inset shows an enlarged view of the yellow rectangle, illustrating irregularly entangled *Girvanella* tubules. (**B**) amoeboidal, lobate, and upward-widening dark thromboids. (**C**) isopachous fibrous cement and blocky cement infilling the interframe spaces. (**D**) angular to subangular intraclasts with well-delineated outlines. (**E**) fine sand-sized, subangular to subrounded peloids. (**F**) silt to fine sand-sized, angular quartz grains. (**G**) coated grains surrounded by microbial components. (**H**) bioclasts (Bc) and micrite (Mc).

To assess annotation accuracy, 10% of the tiles were randomly sampled and cross-checked against manually labeled counterparts, resulting in over 95% consistency. Tiles with undefined or NaN values—typically found near class boundaries or in ambiguous regions—were automatically excluded during preprocessing. Although the exact proportion of removed tiles cannot be reconstructed from the final dataset, this filtering step ensured label consistency and minimized noise in the resulting training data. The final class distribution after filtering is summarized in Table 1, which lists the number of tiles and their proportional representation for each microfacies class. This information guided the stratified sampling strategy for dataset partitioning, ensuring balanced representation across training, validation, and test sets. Class labels follow standard carbonate microfacies classification schemes^13^, and multiple expert reviews were conducted to ensure annotation quality. Detailed class definitions are presented in Table 2, and final integrated labels along with the origin of representative sample images are organized in Table 3. To enhance model training and transparency, Fig. 5 visually illustrates representative tiles with their exact extraction locations within thin section slabs, highlighting the geological source of training data. Figure 6 provides optical microscopy images that capture the morphological diversity of key carbonate components such as *Girvanella* crust, thromboid, and peloid, offering a clearer reference for classification standards. The dataset adheres to the FAIR (Findable, Accessible, Interoperable, Reusable) principles:

- Findable: Each tile and its class label is uniquely tracked via structured file paths and the dataset_labels.csv metadata file. The full dataset is indexed in a DOI-linked Figshare repository.
- Accessible: Publicly released under the CC-BY 4.0 license, available for unrestricted use and download via Figshare.
- Interoperable: Provided in standard CSV (annotations) and PNG (images) formats, compatible with most data processing platforms.
- Reusable: All label definitions, annotation workflows, environment specifications, and automation scripts are fully documented in the Supplementary Information and the accompanying GitHub repository.

**Table 1:** samples after filtering.

Class ID	Class Name	Tile Count	Percentage (%)
0	Skeletal Grainstone	2171	7.94
1	*Girvanella* Boundstone	6900	25.23
2	Peloidal Packstone	129	0.47
3	Micritic Mudstone	811	2.97
4	Thrombolitic Boundstone	1309	4.79
5	Fenestral Wackestone	65	0.24
6	Intraclastic Rudstone	810	2.96
7	Ooidal Grainstone	157	0.57
8	Laminated Microbialite	1402	5.13
9	Dolomitic Mudstone	13591	49.7

**Table 2:** List of slab image files used in this study.

Folder name	Number.png	Grid. png	Rock.png
(cali.) 20150212 HXND 2-3 (1)	(cali.) 20150212 HXND 2-3 (1)_grid & number.png	grid.png	(cali.) 20150212 HXND 2-3 (1)_grid & rock.png
(cali.) 20150212 HXND 2-3 (2)	(cali.) 20150212 HXND 2-3 (2)_grid & number.png	grid.png	(cali.) 20150212 HXND 2-3 (2)_grid & rock.png
(cali.) Unit 1 1-1(1,1)	(cali.) Unit 1 1-1(1,1)_grid & number.png	grid.png	(cali.) Unit 1 1-1(1,1)_grid & rock.png
(cali.) Unit 1 1-2(1,1)	(cali.) Unit 1 1-2(1,1)_grid & number.png	grid.png	(cali.) Unit 1 1-2(1,1)_grid & rock.png
(cali.) Unit 1 1-1(2,1)	(cali.) Unit 1 1-1(2,1)_grid & number.png	grid.png	(cali.) Unit 1 1-1(2,1)_grid & rock.png
(cali.) Unit 1 1-1(3,1)	(cali.) Unit 1 1-1(3,1)_grid & number.png	grid.png	(cali.) Unit 1 1-1(3,1)_grid & rock.png
(cali.) Unit 1 1-1(4,1)	(cali.) Unit 1 1-1(4,1)_grid & number.png	grid.png	(cali.) Unit 1 1-1(4,1)_grid & rock.png
(cali.) Unit 1 1-1(4,2)	(cali.) Unit 1 1-1(4,2)_grid & number.png	grid.png	(cali.) Unit 1 1-1(4,2)_grid & rock.png
(edit) Unit 1 1-1(2)(1,1)	(edit) Unit 1 1-1(2)(1,1)_grid & number.png	grid.png	(edit) Unit 1 1-1(2)(1,1)_grid & rock.png
(edit) Unit 1 1-2(1,1)	(edit) Unit 1 1-2(1,1)_grid & number.png	grid.png	(edit) Unit 1 1-2(1,1)_grid & rock.png
(edit) Unit 1 1-2(4,1)	(edit) Unit 1 1-2(4,1)_grid & number.png	grid.png	(edit) Unit 1 1-2(4,1)_grid & rock.png
(edit) Unit 1 1-5(1)(3,1)	(edit) Unit 1 1-5(1)(3,1)_grid & number.png	grid.png	(edit) Unit 1 1-5(1)(3,1)_grid & rock.png

Each folder contains a set of annotated grid, number, and rock images used for tile generation.

**Table 3:** Classification labels used in the dataset.

Class ID	Class name	Merged label terms	Extracted image
0	intraclast	intraclast (a)	Unit 1 1-5(1)(3,1)
1	*Girvanella* crust	*Girvanella* crust (1)	20150212 HXND 2-3
2	thromboid	thromboid (2)	Unit 1 1-5(1)(2,1) & (3,1)
3	microbial peloid	microbial peloid (3)	Unit 1 1-2(1,1)
4	peloid	peloid (4)	Unit 1 1-5(1)(3,1)
5	coated grain	coated grain (5)	Unit 1 1-5(1)(3,1)
6	bioclast	bioclast (6), bioclast + c	Unit 1 1-2(4,1)
7	quartz	quartz silt to fine sand (7)	20150212 HXND 2-3
8	micrite	micrite, microsparite (8)	Unit 1 1-2(1,1)
9	cement	cement (9)	Unit 1 1-2(1,1)

This table shows the class ID, the label name, the terms merged under each class during label consolidation, the original slab image from which each class was extracted, and the filename of the representative extracted image tile used for visualization (see Fig. 4).

Note: The following labels were annotated during the manual labeling phase but were not included in the final dataset: *Angusticellularia* (e), *Epiphyton* (f), and *Renalcis* (g). These may be incorporated in future expansions of the label set.

All tile generation and annotation procedures were initially implemented using MATLAB-based automation and have been ported and fully validated in Python to support reproducible workflows.

## Usage Notes

This dataset can be applied in various research contexts:

- Training deep learning models for automated carbonate microfacies classification

- Reconstructing paleoenvironmental and ancient marine ecosystems

- Benchmarking and comparing model accuracy in carbonate image analysis

Compared to traditional thin-section examination, this dataset enhances reproducibility and scalability in automated analysis workflows. In particular, it offers a standardized reference for benchmarking deep learning models and supports transfer learning for diverse carbonate microfacies datasets. It can also serve as transfer learning material for other machine learning models^14,15^. **Supplementary Information** provides technical details necessary for reproducing the dataset construction and tile labeling pipeline, including environment setup, workflow steps, folder structure, and variable descriptions.

## Supplementary information

Supplementary Information

## Data Availability

All data supporting this study are available in Figshare (10.6084/m9.figshare.31033825)^12^.

## Code availability

All Python scripts developed for this study are already publicly available without restrictions. The complete codebase, including scripts for automated thin-section tile generation, annotation handling and class assignment, and visualization tools for label integration and validation, is permanently hosted on GitHub (https://github.com/neobassist/girvanella-boundstone-code) under a CC-BY 4.0 license.

The released materials include:

• Python scripts for automated thin-section tile generation

• Python code for annotation handling and class assignment

• Visualization tools for label integration and validation

The full workflow, including a Jupyter Notebook (.ipynb), a Conda environment configuration file (.yml), class label mapping files (label_map.py, label_map.csv), and a detailed README.md with step-by-step instructions, is already openly accessible via GitHub and is intended to ensure long-term accessibility and reproducibility.

## References

[1] Ezaki, Y., Liu, J., Adachi, N. & Yan, Z. Microbialite development during the protracted inhibition of skeletal-dominated reefs in the Zhangxia Formation (Cambrian Series 3) in Shandong Province, North China. *PALAIOS* **32**, 559–571 (2017).

[2] Adachi, N., Ezaki, Y., Liu, J. & Yan, Z. Cambrian through Ordovician reef transitions in North and South China: Changes in reef construction and background geobiological environments. *Palaeogeography, Palaeoclimatology, Palaeoecology* **630**, 111804 (2023).

[3] Wood, R., Zhuravlev, A. Y., Debrenne, F. & Riding, R. Functional biology and evolution of archaeocyathan reefs. *Palaeontology* **35**, 1–17 (1992).

[4] Riding, R. Microbial carbonates: the geological record of calcified bacterial-algal mats and biofilms. *Sedimentology* **47**, 179–214 (2000).

[5] Liu, W. & Zhang, X. *Girvanella*-coated grains from Cambrian oolitic limestone. *Facies* **58**, 779–787 (2012).

[6] Kim, D., Choh, S. J., Liu, W., Zhang, X. & Hong, J. Cambrian Series 2 calcimicrobial crust–cement boundstone in the Yangtze Block, China: A distinctive bioconstruction as a legacy of Precambrian reef evolution. *Sedimentary Geology* **477**, 106804 (2025).

[7] Liu, X. & Song, H. Automatic identification of fossils and abiotic grains during carbonate microfacies analysis using deep convolutional neural networks. *Sedimentary Geology* **410**, 105790, 10.1016/j.sedgeo.2020.105790 (2020).

[8] Koeshidayatullah, A. S., Rahman, A., Reza, R. A., Putra, A. P. & Abdullah, N. Fully automated carbonate petrography using deep convolutional neural networks. *Pure KFUPM Repository* https://pure.kfupm.edu.sa/en/publications/fully-automated-carbonate-petrography-using-deep-convolutional-ne (2020).

[9] Nande, A. & Patwardhan, S. Intelligent identification of carbonate components based on YOLOv5. *Facies* https://link.springer.com/article/10.1007/s10347-024-00694-x (2024).

[10] Vieira de Mello, A., da Silva, M. R. & dos Santos, R. Deep mineralogical segmentation of thin section images using CNN + QEMSCAN maps. *arXiv preprint* arXiv:2505.17008 https://arxiv.org/abs/2505.17008 (2025).

[11] Al-Fahdi, A. *et al*. *Lithofacies and microfacies and depositional environment model of the Cenozoic carbonate platform: an example from the Upper Jafnayn Formation of Jafnayn area in north-east Oman*. *Arabian Journal of Geosciences* **17**(12), 320, 10.1007/s12517-024-12094-0 (2024).

[12] Choi, S. Y. *et al*. High-resolution annotated dataset of Girvanella boundstone microfacies from the Xiannüdong Formation, China. Figshare, 10.6084/m9.figshare.31033825 (2026). 10.1038/s41597-026-06958-1 PMC13083876 41786785

[13] Flügel, E. Microfacies of Carbonate Rocks: Analysis, Interpretation and Application. Springer (2010).

[14] Adachi, N., Ezaki, Y. & Liu, J. Cambrian microbial reefs thriving in an oxygen-stratified early marine environment. *Geology* **32**, 881–884 (2004).

[15] Li, K., Song, J., Yan, H., Liu, S. & Yang, D. Carbonate microfacies classification model based on dual neural network: a case study on the fourth member of the upper Ediacaran Dengying Formation in the Moxi gas field, Central Sichuan Basin. *Arabian Journal of Geosciences* **15**, 1773 (2022).

