# Supplementary material for: High-resolution Annotated Dataset of Girvanella Boundstone Microfacies from the Xiannüdong Formation, China
Source: Sci Data. 2026 Mar 5;13:611. doi: 10.1038/s41597-026-06958-1 (PMC13083876; doi:10.1038/s41597-026-06958-1)
Supplement: Supplementary file 1 — Supplementary Information [file 41597_2026_6958_MOESM1_ESM.docx]

# Supplementary information: Python Workflow for Thin Section Tile Extraction

## 1. Python Environment Setup

To replicate the preprocessing and tile annotation process described in this study, we provide two methods to set up the Python environment: manual installation using Conda or automatic reproduction using a YAML configuration file.

Option 1. Manual setup with Conda
***conda create -n thinsection_tiles python=3.9.19 -y
conda activate thinsection_tiles
conda config --set solver classic
conda config --add channels defaults
conda install -c conda-forge ipykernel jupyter opencv numpy pandas --solver classic -y
python -m ipykernel install --user --name thinsection_tiles --display-name "thinsection_tiles"***

Option 2. YAML-based environment replication
***conda env create -f thinsection_tiles.yml
conda activate thinsection_tiles
python -m ipykernel install --user --name thinsection_tiles --display-name "thinsection_tiles"***

Note: These commands are compatible across macOS, Linux, and Windows platforms. Make sure the 'thinsection_tiles.yml' file is placed in the working directory before executing the commands.

Additional packages such as matplotlib, scikit-learn, and seaborn are required for dataset visualization and evaluation metrics.
***conda install matplotlib scikit-learn seaborn -y***

## 2. Tile Extraction Workflow Overview

The Python-based workflow replicates the tile-based preprocessing previously developed in MATLAB. The Jupyter notebook 'thinsection_tile_DATASET_workflow_final.ipynb' performs tile slicing, annotation, filtering, and dataset construction.

## 3. Input and Output Folder Structure

After executing the workflow, the following folders are generated:
- DAT/LV1/{sample_name}/ : Input folder containing 'rock.png', 'number.png', and 'grid.png' per sample
- FIG/LV1/{sample_name}/ : Stores all generated tiles per sample, regardless of label (NaN included)
- DATASET_MERGE/ : Merged tiles that passed annotation
- DATASET/:
 ├── dataset_train/ : Training tiles
 ├── dataset_val/ : Validation tiles
 ├── dataset_test/ : Test tiles
 └── dataset_labels.csv : CSV mapping each tile path to its label

## 4. Summary of Python Workflow Steps

1. Load rock.png, number.png, and grid.png from each sample directory in DAT/LV1/{sample_name}/.
2. Apply the grid image to subdivide rock.png into tiles of 114×114 pixels.
3. For each tile, compute the sum of green-channel pixel values in the corresponding region of number.png.
4. Save all tiles to FIG/LV1/{sample_name}/, regardless of class information.
5. If the computed sum corresponds to a valid class ID in label_map, copy the tile to DATASET_MERGE/{class}/.
6. Generate a metadata file, dataset_labels.csv, which records the image path, class ID, tile position, and sample information.

**Note**:

Class annotations are embedded in the **green channel** of number.png, and the **pixel sum** within each tile determines the assigned class via label_map. The classes *Angusticellularia*, *Epiphyton*, and *Renalcis* were present in early-stage annotations but excluded from the final classification due to insufficient representation in the dataset.

## 5. Description of Key Python Variables

- sample_folders: List of sample folders under 'DAT/LV1/'
- grid_file: Grid image file used to define tile boundaries
- rock_file: Raw thin section image (rock.png)
- number_file: Annotation mask encoding class information (number.png)
- tile_size: Resolution of each tile (114×114 pixels)
- interval: Central crop interval (104 pixels)
- tile_output_dir: Output directory under 'FIG/LV1/' where all tiles are stored by sample
- total_tiles: Global counter for sequentially naming all tiles
- image_sum_values: List of pixel sum values (green channel) used for classification
- label_map: Mapping between pixel sum and class index
- merged_dataset_dir: Directory 'DATASET_MERGE/' where annotated tiles are copied
- split_output_dir: Output directory for split dataset
- split_ratio: Train/Val/Test split ratios (e.g., 0.8, 0.1, 0.1)
- random_seed: Random seed to ensure reproducibility in dataset splitting
- dataset_csv_path: Path to the output CSV file listing tile paths and labels
